# Measuring adaptive expertise and adaptive performance in (becoming) healthcare professionals: a scoping review of measurement instruments

**DOI:** 10.1007/s10459-025-10413-y

**Published:** 2025-01-30

**Authors:** Elske Hissink, Els Pelgrim, Loek Nieuwenhuis, Lotte Bus, Wietske Kuijer-Siebelink, Marieke van der Schaaf

**Affiliations:** 1 Radboud University Nijmegen Medical Centre, Nijmegen, The Netherlands; 2 Avans University of Applied Sciences, Breda, The Netherlands; 3 HAN University of Applied Sciences, Arnhem, The Netherlands; 4 University Medical Center Utrecht, Utrecht, The Netherlands

**Keywords:** Adaptive expertise, Adaptive performance, (Becoming) healthcare professionals, Measurement instruments, Operationalizing adaptive expertise and adaptive performance

## Abstract

Professional work in healthcare is increasingly disruptive, requiring professionals to be flexible and adaptable. Research on adaptive expertise and adaptive performance in healthcare has grown, and operationalisation and measurement of these concepts are crucial to meet professionals’ evolving needs. This study provides an overview of measurement instruments for adaptive expertise and adaptive performance in (becoming) healthcare professionals, including an evaluation of their operationalisations and the amount of evidence supporting their quality. A scoping review was conducted, including an analysis of operationalisations and the amount of evidence supporting the quality of instruments, based on the criteria outlined in the *Standards for Educational and Psychological Testing* (2014). Seventeen articles were included, comprising nineteen measurement instruments, three of which were specifically developed for the healthcare domain. The instruments, categorized into six types, varied in the nature and volume of evidence supporting their quality. Analysis of the operationalisations led to a clearer understanding of the concepts, with themes emerging around adaptive expertise and adaptive performance. The study reveals a dominance of self-evaluation and job requirement instruments, while other methods, such as design scenarios, mixed-methods instruments, and collegial verbalization, are underrepresented. Instruments developed specifically for healthcare need further validation and reliability testing. The categorization of subscales into 13 themes provides further clarification of the concepts and suggestions for future research. Instruments to measure adaptive expertise and adaptive performance are limited and vary in conceptualisation, operationalisation and quality. Further research is needed to improve the validity and reliability of healthcare-specific instruments.

**Supplementary Information:**

The online version contains supplementary material available at 10.1007/s10459-025-10413-y.

## Introduction

In healthcare, professionals must be prepared to face the demands of an ever-changing professional landscape and work environment, including solving novel problems and handling ambiguous and complex medical situations (Mylopoulos et al., 2022). This requires that they both apply their extensive knowledge base and rapidly acquire additional skills as needed (Mylopoulos et al., 2018).

The theoretical framework of adaptive expertise helps to understand and explore how the challenges faced by healthcare professionals and becoming healthcare professionals, such as medical students, can be addressed (Mylopoulos et al., 2022). These challenges manifest in both cognitive abilities and the precise execution of technical skills (Dall Jensen et al., 2022). As novices, students have limited opportunities to develop expertise and have yet to cultivate adaptive expertise. However, building this capacity is essential to prepare them for the complexities of future healthcare challenges.

In 1986, Hatano and Inagaki (1986) first introduced and conceptualised the term adaptive expertise. They positioned adaptive expertise in addition to routine expertise. Routine expertise represents the knowledge and skills that enable efficiency to professionally perform in routine situations. Adaptive expertise includes knowledge and skills that support both efficiency during professional work, but also the capability to apply flexible problem-solving approaches and generate novel solutions when unusual circumstances occur. As adaptive expertise is not automatically visible, given that knowledge and skills can be tacit, the concept of adaptive performance is mentioned in the literature (Pelgrim et al., 2022). A considerable amount of work has been undertaken with the aim of precisely defining these concepts. Although both terms seem related, a lack of conceptual clarity exists regarding the precise definitions of these terms (Cupido et al., 2022). Pelgrim and colleagues found in their metareview that there is little consensus regarding the exact terminology used to describe the concepts of adaptive expertise and adaptive performance, with different terms being used in various publications (Pelgrim et al., 2022). Despite these differences, most conceptualisations have in common that adaptive performance is best referred to as the visible outcome of adaptive expertise, which is triggered by (contextual) change in tasks or environments.

Given the increased attention to adaptive expertise and adaptive performance in health professions education research, further conceptualisation, operationalisation and measurement of these concepts needs attention (Pelgrim et al., 2022). There is currently no comprehensive overview of available instruments for measuring adaptive expertise and adaptive performance in health professions education. Bohle Carbonell et al. already recognized the need for an overview of instruments, scales and items employed to measure adaptive expertise in 2014, and added that measuring adaptive expertise is difficult because characteristics of this concept are not clearly defined (Bohle Carbonell et al., 2014). In their 2021 scoping review, Kua et al. searched for existing evidence in conceptual frameworks, development, and measurement for adaptive expertise. They report two validated instruments for measuring adaptive expertise but note that there are still significant gaps in the development and validation of such tools. They emphasize the need for suitable measurement instruments both within and outside of clinical practice in healthcare (Kua et al., 2021, p. 1).

To ensure accurate measurement of adaptive expertise and adaptive performance, it is essential to identify appropriate instruments that meet central quality criteria like validity, reliability and fairness. The Standards for Educational and Psychological Testing, also known as ‘the Standards’ (American Educational Research Association, American Psychological Association, & National Council on Measurement in Education, 2014), describe these quality criteria and are considered as best practice in the field of psychometrics (Streiner et al., 2008). The Standards are used in this study to evaluate the amount of evidence provided by authors regarding the central quality criteria of their instruments. According to the Standards, validity is the most fundamental consideration in developing tests and evaluating tests. Validity refers to “the degree to which evidence and theory support the interpretations of test scores for proposed uses of tests” (AERA, APA & NCME (Eds.), 2014, p. 11). The second important criterion is reliability. The Standards use the term reliability, which stands for precision to “denote the more general notion of consistency of the scores across instances of the testing procedure”, and the term reliability coefficient to “refer to the reliability coefficients of classical test theory” (AERA, APA & NCME (Eds.), 2014, p. 33). The third criterion mentioned is fairness which addresses “the importance of fairness as a fundamental issue in protecting test takers and test users in all aspects of […] test, testing, and test use” (AERA, APA & NCME (Eds.), 2014, p. 49).

Since the need for adaptive expertise and adaptive performance is seen in all kinds of professions that shape future society, including industry, government, the military, and healthcare (Pusic et al., 2022; Stoffels et al., 2022), it is anticipated that instruments for assessing adaptive expertise and adaptive performance can be found across diverse domains. A broad perspective is required due to the potential for cross-disciplinary knowledge exchange among diverse domains, and the demand for adaptive expertise among healthcare professionals who encounter problems necessitating interdisciplinary solutions.

A comprehensive and interdisciplinary overview of measurement instruments would be beneficial to researchers, educators, and human resource development (HRD) professionals in healthcare. In the first place, this would be beneficial for gaining a deeper and more comprehensive insight into how various authors operationalise adaptive expertise and adaptive performance, as well as what these concepts entail in practice. In other words, what do adaptive expertise and adaptive performance "look like" in real-world settings? Furthermore, insight in the amount of evidence provided for the quality of instruments would offer information to guide the selection of instruments to measure the concepts in (becoming) healthcare professionals and to eventually support the development of these abilities.

## Research questions

The study aims to provide an overview of existing measurement instruments for adaptive expertise and adaptive performance, insight in the used operationalisations and concepts and a description of the amount of evidence of the central quality criteria. The scope includes instruments for workplace and educational contexts of professionals, aimed at summative and formative assessment purposes.

A scoping review (Tricco et al., 2018) was conducted, providing a preliminary assessment of the size, scope, and extent of research literature, including ongoing studies (Grant & Booth, 2009). The review was guided by the following research questions:

1. What instruments exist to measure adaptive expertise and adaptive performance, and which are designed for the healthcare domain?
2. How are adaptive expertise and adaptive performance operationalised in the instruments, and what are the implications of these operationalisations for their conceptualisation?
3. What is the amount of evidence for the quality of the instruments?

## Method

The scoping review was conducted in June 2023, following the updated PRISMA-ScR guideline for reporting scoping reviews (Tricco et al., 2018). Table 1 shows the keywords used. In feature 2, in addition to the terms expertise and performance, the term competence is included, because the amalgam of knowledge, skills, and attitudes is also denoted with the term competence (Eraut, 1994).

**Table 1:** Keywords used in the search

Feature 1	Terms related to adaptivity
	adaptiv* or adapta* (within one word of the terms of feature 2)
Feature 2	Terms related to expertise
	expertise OR performance OR competence
Feature 3	Terms related to instruments
	measur* OR assess* OR interview* OR observation* OR questionnaire OR instrument
	OR checklist OR tool OR survey OR scale OR test*

International peer-reviewed articles presenting the keywords in title, abstract and/or author keywords (i.e. keywords that the authors have appended themselves to their article, instead of the editors of a journal) were obtained from the databases of Web of Science and Scopus, using the underlying databases mentioned in Appendix 1. These databases were chosen based on the access they provide to high quality research in the domains of Learning and Development (HRD/HRM) and Education across different disciplinary fields. The timespan was limited to publications released between 1986 (emergence of the concept of adaptive expertise) and June 2023. The search strategy used was developed in an iterative process by EH, EP, LN, WK and MvdS, in consultation with a research librarian.

### Screening and selection of articles

To limit and specify the search, the following inclusion criteria for title abstract screening were developed:

1. The main focus of the article is a measurement instrument or the development of a measurement instrument that measures adaptive expertise or adaptive performance;
2. The article features a concise discussion pertaining to the quality attributes of the instrument.

The selected publications were managed and filtered for duplicates in Endnote™ citation manager resulting in a first output of 163 unique articles. Deduplicated publications were exported and analysed on in- and exclusion criteria using Rayyan (Ouzzani et al., 2016). EH, LN and EP conducted the screening process, using a two-step approach. Titles and abstracts of all articles were independently reviewed by two researchers (EH and LN), and if there was conflicting outcome concerning the inclusion or exclusion of an article (in 12% of the articles), a third researcher (EP) reviewed the title and abstract. The article was then discussed and a decision was made. This process resulted in an output of 32 included articles.

Inclusion criteria were then further sharpened which led to the following additional inclusion criteria for full text screening:

1. The article relates to at least one of the four research questions;
2. The language of the full text is English;
3. The items of the instrument are accessible, either within the article, online, or through another publication;
4. The focus of the article is on healthy individual (becoming) professionals, i.e. not on the team or organisational level;
5. Part of the article describes the quality of the measurement instrument.

In the second phase of the inclusion, two researchers (EH and LN) reviewed the full text of the 32 articles. In case of conflict concerning inclusion or exclusion (in 22% of the articles) between the two researchers, the third researcher (EP) again reviewed the article. After a discussion between the three researchers, based on the proposal of each of the three researchers and their arguments for inclusion or exclusion, a final decision for inclusion or exclusion was made.

Of the 32 articles reviewed, 15 were excluded, leaving 17 articles that describe 19 measurement instruments. Of the 15 articles that did not meet the inclusion criteria, two articles were written in Turkish or German; nine articles did not provide an instrument but instead only described a theoretical background, guiding principles, operationalisations or combinations of instruments useful to develop measurement instruments; one article described an instrument measuring the concept of adaptive expertise mainly at organisational level; one article included an instrument regarding individuals with specific characteristics (intellectual disabilities) and two articles only described the utilization of an instrument, without further explanation.

Based on one of our inclusion criteria (the items of the instrument are accessible) we should have excluded the Job Adaptability Inventory (JAI), since the items of Pulakos’ JAI have not been publicly disclosed. However, since several later instruments are based on this instrument, we decided to include the JAI in the 17 articles.

**Fig. 1:**
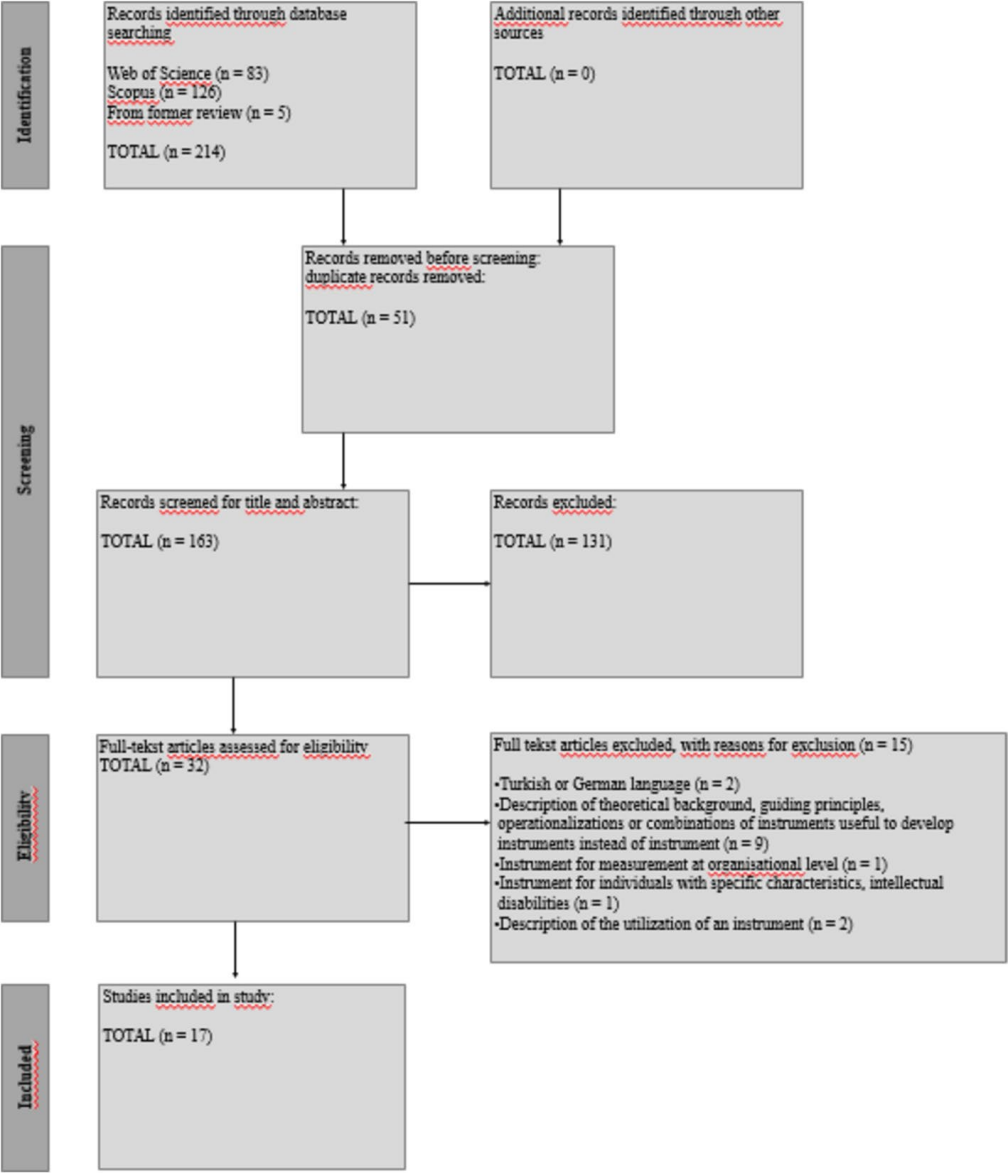
Identification of the articles: PRISMA Flow Diagram for the scoping review process (Peters et al., 2020)

Figure 1 illustrates the identification of the articles.

### Positionality

This research project focused on measuring adaptive expertise and adaptive performance in (becoming) healthcare professionals. The entire research project lasted 1.5 years. During this period, there were regular consultations with the entire team regarding the research approach, search methodology, search strings, and the data analysis. The data analysis was carried out by three researchers who had bi-weekly meetings. Throughout this period, the entire team provided background input, once every two months. Assumptions and perspectives were questioned and the data collection, analysis, and interpretation were shaped. During the article writing phase, the contact became more frequent, approximately once every two weeks for those directly involved in the writing process. To be able to analyse all nineteen measurement instruments, working in a multidisciplinary team which included various research orientations and different disciplinary backgrounds was deliberately chosen. The team’s expertise included educational science (EH, EP, LN, LB and WK), health professions education (EH, EP, WK and MvdS) and industrial innovation education (LN). In the research team researchers bring in psychological perspectives, e.g. creativity, interpersonal communication, and expert perception (MvdS) and sociological perspectives, e.g. change processes in organizations (LN and MvdS). The multi-disciplinarity of the research team was valuable during the discussions, as it provided an opportunity to integrate knowledge and expertise from different professional backgrounds.

### Data analysis

#### Extracting general information

The first step of the data analysis was to extract general information from the included publications, such as: year of publication; name and type of measurement instrument; conceptualisation; operationalisation; literature and possible previous instruments that the instrument is based on; goal of the instrument; target group of the instrument and potential applications for use in various domains.

#### Operationalisation and conceptualisation

The second step of the data analysis involved discussing the operationalisations of the instruments to contribute to the ongoing debate about conceptualizing and clarifying the terms adaptive expertise and adaptive performance. The main goal was to refine the descriptions of both concepts. Three researchers (EH, EP and LN) reviewed all subscales (also referred to by the authors as dimensions, factors, scales and concepts), and the operationalisations used by the authors. The objective was to assess the extent of overlap in language or content between the subscales and whether the authors' operationalisation provided deeper insights into the conceptualisation of adaptive expertise and adaptive performance. All subscales were studied individually, then discussed in pairs (EH and EP, EH and LN) and finally with the full research team. This process resulted in a categorization of themes representing either adaptive expertise or adaptive performance, providing a representative view of operationalisation that the authors used in measuring adaptive expertise and adaptive performance.

#### Evaluating evidence for the central quality criteria

The third step of the data analysis involved evaluating the extent to which the quality of the instruments was described. To assess this, the central criteria of the Standards (2014) were used: validity, reliability and fairness in testing. An evaluation was conducted on the information provided by the authors, categorizing the evidence for each aspect of quality as either limited evidence, moderate evidence or strong evidence.

We categorized evidence as limited if no relevant information was provided in the publication or if the information regarding the quality criteria was incomprehensible or incomplete. Evidence was categorized as moderate if at least one piece of relevant information was available. This could include, for example, a traceable literature review, statistical analysis, one piece of evidence regarding reliability, and clear documentation of test design, validation, development, administration, and scoring procedures to minimize construct-irrelevant variance and promote valid score interpretations for all examinees in the intended population. Evidence was categorized as strong if multiple pieces of evidence supported the quality criteria and each cluster of validity, reliability, and fairness was thoroughly elaborated.

The two primary investigators (EH and LN) independently evaluated all 19 measurement instruments published in 17 journal articles. EH and LN thoroughly discussed the instruments during eight sessions, each focusing on one to three instruments. They documented decisions made during these sessions. This approach allowed time between sessions for thorough article review, and during the sessions, they utilized arguments from previous discussions. While discussing the criteria, we added a brief description of each instrument based on the two criteria of part III of the Standards (2014), namely the testing applications: 1: Workplace testing and credentialing, 2: Educational testing and assessment. This brief description provided us with a summary of each instrument, including conceptualisation and the domain each instrument is used in.

## Results

### Overview of the instruments

The 17 articles that were found to meet the inclusion criteria provide descriptions of 19 measurement instruments that can be grouped into six instrument types:

I. *Job evaluations* aim to assess professional’s adaptive performance requirements for a job;
II. *Self-evaluations* aim to self-evaluate the adaptive expertise or adaptive performance of (becoming) professionals;
III. *Supervisor evaluations* used by supervisors to evaluate the adaptive expertise or adaptive performance of (becoming) professionals;
IV. *Design scenarios* present individual participants with a brief description of a realistic, complex, open-ended design challenge. Participants respond to open questions on how they would solve the challenge;
V. *Mixed methods* a mix of diverse instruments to evaluate the adaptive expertise or adaptive performance of (becoming) professionals;
VI. *Collegial verbalizations* verbalization protocols that are utilized to validate collected verbal data on recorded material from naturalistic work environments. Colleagues of employees watch videos and verbalize on the employees’ behaviour.

Table 2 offers a summary of the instruments, including instrument type, information on the specific context in which each instrument is employed, concepts and operationalisations of adaptive expertise or adaptive performance, any prior instruments that may have influenced its development, as well as the individuals or groups responsible for developing the instrument. The order of the instruments is determined by the instrument type, beginning with the Job Adaptability Inventory, as numerous subsequent instruments derive from it.

**Table 2:** Overview of instruments measuring adaptive expertise or adaptive performance

	Instrument name	Summary description of the measured concept and context (educational or workplace setting)	Operationalisation: subscales	Instrument developed by / Authors	Instrument derived from	Year of publication
*Instrument type: Job evaluation*						
#1	Job Adaptability Inventory (JAI)	The instrument is used to diagnose a job's adaptive performance requirement in workplace settings (variety of different job types, mainly military jobs)	8-dimension taxonomy: (1) handling emergencies or crisis situations; (2) handling work stress; (3) solving problems creatively; (4) dealing with uncertain and unpredictable work situations; (5) learning work tasks, technologies, and procedures; (6) demonstrating interpersonal adaptability; (7) demonstrating cultural adaptability; (8) demonstrating physically oriented adaptability 68 items on 5-point Likert scale	Pulakos et al. (2000)	Instrument derived from the Job performance model developed by Campbell, McCloy, Oppler, and Sager (1993)	2000
#2	Dutch Adaptability Dimensions And Performance Test (D-ADAPT): Work ADAPT	The instrument is used to measure the adaptability demands of jobs in workplace settings (military and civil domain)	6 dimensions adaptability dimensions: (1) handling crisis situations, (2) solving difficult problems, (3) culturally demanding situations, (4) physically demanding circumstances, (5) handling work stress, (6) interpersonal interactions 31 items on a 5-point Likert scale	Oprins et al. (2018)	Instrument derived from the Job Adaptability Inventory by Pulakos et al. (2000) and the I-ADAPT-M	2018
*Instrument type: Self-evaluation*						
#3	“An adaptive expertise Likert-scale” (instrument name is not specified)	The instrument is used to measure self-reported qualities of adaptiveness in three target populations in the engineering educational settings (higher education: biomedical engineering freshmen, senior and faculty)	4 main constructs: (1) multiple perspectives; (2) metacognition; (3) goals and belief; (4) epistemology 42 items on 6-point Likert scale	Fisher et al. (2001)	Instrument derived from previous research (cognitive science literature) but not from a specific previous instrument	2001
#4	Job Adaptability Inventory (JAI)	The instrument is used to measure the self-reported eight adaptive performance dimensions of employees in workplace settings (military personnel) The instrument is also used for supervisor evaluation in workplace settings (military personnel)	8-dimension taxonomy: (1) handling emergencies or crisis situations; (2) handling work stress; (3) solving problems creatively; (4) dealing with uncertain and unpredictable work situations; (5) learning work tasks, technologies, and procedures; (6) demonstrating interpersonal adaptability; (7) demonstrating cultural adaptability; (8) demonstrating physically oriented adaptability	Pulakos et al. (2002)	Instrument derived from the previous Job Adaptability Inventory by Pulakos et al. (2000)	2002
#5	“A new scale” (instrument name is not specified)	The instrument is used to measure self-reported adaptive performance in workplace settings (a variety of job contexts)	5 dimensions of adaptive performance: (1) creativity; (2) reactivity in the face of emergencies or unexpected circumstances; (3) interpersonal adaptability; (4) training and learning effort; (5) managing work stress 19 items on a 7-point Likert scale	Charbonnier-Voirin et al. (2012)	Instrument derived from the Job Adaptability Inventory by Pulakos et al. (2000)	2012
#6	Adaptive Expertise Inventory	The instrument is used to measure self-reported adaptive expertise in a wide range of workplace settings (mostly professional, scientific, technical and educational areas)	2 dimensions measuring adaptive expertise (domain-specific skills and innovative skills) 10 items on a 5-point Likert scale	Bohle Carbonell et al. (2016)	Instrument derived from instruments of Fisher et al. (2001) and van der Heijden (2000)	2016
#7	Individual measure of AP; team level AP	The instrument is used to measure self-reported individual adaptive performance in workplace settings (different domains)	2 factors: Problem solving oriented factor and learning work tasks factor 6 items on a 7-point Likert scale	Marques-Quinteiro et al. (2015)	Instrument derived from the Job Adaptability Inventory by Pulakos et al. (2000)	2015
#8	Fleadapt scale	The instrument is used to measure self-reported adaptability in workplace settings (frontline employees)	7 dimensions of FLE adaptability: (1) interpersonal adaptability, (2) service offering adaptability, (3) political adaptability, (4) social aspects of adaptability, (5) physical aspects of adaptability, (6) group adaptability, (7) organization adaptability 41 items on a 7-point Likert scale	Sony et al. (2015)	Instrument derived from the seven dimensions for FLE adaptability (Sony et al. 2014)	2015
#9	“Measure adopted from previous studies” (instrument name is not specified)	The instrument is used to measure self-reported adaptive expertise of employees in workplace settings (different enterprises with a commercial recognition)	4 dimensions: (1) solving problems creatively, (2) dealing with uncertain or unpredictable work situations, (3) learning new tasks, technologies and procedures, (4) handling work stress 8 items on a Likert scale	Stanczyk et al. (2017)	Instrument derived from the Job Adaptability Inventory by Pulakos et al. (2000)	2017
#10	Dutch Adaptability Dimensions And Performance Test (D-ADAPT): self-ADAPT	The instrument is used to measure self-reported adaptability competency of employees in workplace settings (military and civil domain)	6 dimensions adaptability dimensions: (1) handling crisis situations, (2) solving difficult problems, (3) culturally demanding situations, (4) physically demanding circumstances, (5) handling work stress, (6) interpersonal interactions 31 items on a 5-point Likert scale	Oprins et al. (2018)	Instrument derived from the Job Adaptability Inventory from Pulakos et al. (2000) and the I-ADAPT-M	2018
#11	Revised Adaptive Expertise Survey	The instrument is used to measure students’ self-reported adaptive expertise in educational settings, to understand the potential impact of curricular requirements on students (post-secondary students in the health, business, science, engineering and social science field)	3 concepts: Domain agility, Self-assessed innovative practice, Orientation to innovation 13 items on a 7-point Likert-scale	Ferguson et al. (2018)	Instrument derived from instruments of Fisher et al. (2001); van der Heijden (2000); Charbonnier-Voirin et al. (2012); Bohle Carbonell et al. (2016), and the General Self-Efficacy Scale (GSES-12)	2018
#12	Individual Work Performance Questionnaire	The instrument is used to measure self-reported individual work performance in workplace settings (different managerial types of jobs: senior manager, middle manager, group leader)	3 factors (work performance types): (1) task performance, (2) contextual performance, (3) counterproductive work behaviour. Items 6, 8 and 9 emerged as a factor of its own, roughly representing adaptive work performance 18 items on a 5-point Likert scale	Dåderman et al. (2020)	Instrument derived from the Dutch version of IWPQ (Koopmans et al. 2013)	2020
#13	The Adaptability Scale	The instrument is used to assess self-reported employee adaptability in workplace settings (different employees in several fields with lower, higher vocational training and university)	3 factors (representing employee adaptability): (1) cognitive adaptability, (2) behavioural adaptability, (3) affective adaptability 10 items on a 5-point Likert scale	van Dam et al. (2021)	Instrument derived from previous research but not from a specific previous instrument	2021
#14	Spanish adaptation of Charbonnier-Voirin and Roussel’s (2012) scale for measuring adaptive performance	The instrument is used to assess self-reported employee adaptability in workplace settings (public sector)	Five dimensions: (1) Managing Work Stress; (2) Training Effort; (3) Interpersonal Adaptability; (4) Reactivity in the Face of Emergencies, (5) Creativity 19 items on a 7-point Likert scale	Gorostiaga et al. (2022)	Instrument derived from the instrument of Charbonnier-Voirin et al. (2012)	2022
*Instrument type: Supervisor evaluation*						
#15	Job Adaptability Inventory (JAI)	The instrument is used by supervisors to measure adaptive performance of employees in workplace settings (military personnel) The instrument is also used for self-evaluation in workplace settings (military personnel)	8-dimension taxonomy: (1) handling emergencies or crisis situations; (2) handling work stress; (3) solving problems creatively; (4) dealing with uncertain and unpredictable work situations; (5) learning work tasks, technologies, and procedures; (6) demonstrating interpersonal adaptability; (7) demonstrating cultural adaptability; (8) demonstrating physically oriented adaptability	Pulakos et al. (2002)	Instrument derived from the previous Job Adaptability Inventory from Pulakos et al. (2000)	2002
*Instrument type: Design scenarios*						
#16	Design scenarios	The instrument is used to assess adaptive expertise within the context of students' regular academic coursework in the educational setting (higher education: biomedical engineering design)	Three questions to examine: (1) efficiency or ability to devise an appropriate response; (2) innovation or ability to consider important facets of a problem; (3) confidence / cautiousness	Walker et al. (2006)	Instrument derived from previous research but not from a specific previous instrument	2006
*Instrument type: Mixed method*						
#17	“Model for assessing teacher’s AE” (instrument name is not specified)	The publication proposes a model of adaptive expertise needed for teachers to successfully deliver NGSS-informed computer supported complex systems curricula in high school science classrooms The instrument is used to assess students’ adaptivity in educational settings (high school level; science teachers)	3 essential characteristics: (1) flexibility; (2) deeper level understanding; (3) deliberate practice Classroom observations, year-end teacher interviews, teacher reflections, researcher focus group interviews. Analysing Adaptive Expertise enactments: categorizing teachers’ Adaptive Expertise (high, medium, low)	Yoon et al. (2019)	Instrument derived from previous research and mixed methods but not from a specific previous instrument	2019
#18	Acquiring / creating teacher profiles on adaptive teaching expertise (instrument name is not specified)	The study shows a new way to create systematic profiles of adaptive teaching experience in educational settings (elementary science teachers)	Instruments: vignettes, interviews, reflections	Suh et al. (2023)	Instrument derived from the instrument of Fisher et al. (2001)	2023
*Instrument type: Collegial verbalization*						
#19	Collegial Verbalization	The instrument is used to identify individual differences which are an important source in understanding AE in workplace settings (train-drivers, high-speed ferry drivers, train dispatchers, long-haul truck drivers and intensive care unit nurses)	Verbalization protocols that are utilized to validate collected verbal data on recorded material from naturalistic work environments. Colleagues of employees watch videos and verbalize on the employees’ behaviour	Axelsson et al. (2018)	Instrument derived from previous research but not from a specific previous instrument	2018

Most of the 19 measurement instruments in Table 2 can be applied in various domains. However, some are specifically developed for and used in healthcare settings, namely the Adaptive Expertise Likert-scale (#3), the Revised Adaptive Expertise Survey (#11), and Design Scenarios (#16). However, both the conceptualisation and operationalisation of the concepts in these instruments are not specifically tailored to the healthcare domain but are generic in nature.

The majority of the instruments (14 in total) are developed for and specifically used in workplace settings. These include instruments for job evaluation, self-evaluation, supervisor evaluation, and collegial verbalization (#1, #2, #4, #5, #6, #7, #8, #9, #10, #12, #13, #14, #15 and #19). Some of the self-evaluation instruments are designed specifically for educational settings in healthcare (#3 and #11). Other instruments developed specifically for educational settings include design scenarios (used in educational healthcare settings) (#16), and mixed method instruments (#17 and #18).

Several instruments are derived from the Job Adaptability Inventory by Pulakos et al. (2000), but other instruments are also mentioned as precursors to newly developed ones. Some authors indicate that their instrument is based on literature reviews but do not specify this further. Certain instruments use nearly identical items for job evaluation, self-evaluation and/or supervisor evaluation. The theoretical foundation and how this relates to perspectives on work, adaptive expertise and adaptive performance is frequently not elaborated upon.

### Conceptualisations and operationalisations

A comparison of the conceptualisations of adaptive expertise and adaptive performance shows that the definitions of the concepts vary across the articles. The theoretical background and frameworks of the instruments exhibit variety and the literature study in the articles is not always traceable. Similar to Pelgrim et al. (2022) and Cupido et al. (2022), we encountered inconsistency in the utilization of terminology and there is no singular, consistent conceptualisation of adaptive expertise or adaptive performance. This results in various operationalisations in the measurement instruments. In this study we used these operationalisations as a starting point for conceptualizing. We were able to recategorize all subscales (the operationalisations) into 13 themes that are related either to adaptive expertise (6 themes) or to adaptive performance (7 themes). We found that the subscales associated with adaptive expertise mainly pertain to *aspects of a person*, specifically their *competencies or traits*. The subscales related to adaptive performance mainly refer to *behaviours or actions* that a professional might demonstrate, related to their *work or tasks*. The classification derived from the analysis is presented in Table 3.

**Table 3:** Themes related to adaptive expertise or adaptive performance

Themes related to adaptive expertise Aspects of a *person*	Themes related to adaptive performance Aspects of* work or tasks*
1. Epistemology / epistemic orientation	1. Handling emergencies or crisis situation
2. Innovative skills	2. Handling work stress
3. Domain specific skills	3. Solving problems creatively
4. Flexibility	4. Dealing with uncertain and unpredictable work situations
5. Metacognition	5. Learning work tasks, technologies and procedures
6. Goals and beliefs	6. Demonstrating interpersonal and cultural adaptability
	7. Demonstrating physical oriented adaptability

In *Supplementary Material_File 1_Analysis of the Subscales*, each subscale from the original instruments is listed under the corresponding theme in our classification.

### Evidence for the quality of the instruments

All instruments were reviewed to assess the extent to which the instrument developers provide evidence in their scientific articles for the central quality criteria validity, reliability, and fairness. Table 4 presents an overview of the amount of evidence for the quality of the measurement instruments.The order of the instruments is again determined by the instrument type.

**Table 4:** Evidence for Validity, Reliability and Fairness of testing of the measurement instruments

Instrument	Authors	Validity	Reliability	Fairness
*Instrument type: Job evaluation*				
Job Adaptability Inventory (JAI) (2000)	Pulakos et al. (2000)	Strong evidence	Strong evidence	Strong evidence
Dutch Adaptability Dimensions And Performance Test (D-ADAPT): Work ADAPT (2018)	Oprins et al. (2018)	Moderate evidence	Moderate evidence	Moderate evidence
*Instrument type: Self-evaluation*				
“An adaptive expertise Likert-scale” (2001)	Fisher et al. (2001)	Limited evidence	Limited evidence	Moderate evidence
Job Adaptability Inventory (JAI) (2002)	Pulakos et al. (2002)	Strong evidence	Moderate evidence	Moderate evidence
“A new scale” (2012)	Charbonnier-Voirin et al	Strong evidence	Moderate evidence	Strong evidence
Adaptive Expertise Inventory (2015)	Bohle Carbonell et al. (2012)	Strong evidence	Moderate evidence	Moderate evidence
Individual measure of AP; team level AP (2015)	Marques-Quinteiro et al. (2015)	Moderate evidence	Limited evidence	Limited evidence
Fleadapt scale (2015)	Sony et al. (2015)	Strong evidence	Strong evidence	Strong evidence
“Measure adopted from previous studies” (2017)	Stanczyk et al. (2017)	Limited evidence	Limited evidence	Limited evidence
Dutch Adaptability Dimensions And Performance Test (D-ADAPT): self-ADAPT (2018)	Oprins et al. (2018)	Moderate evidence	Moderate evidence	Moderate evidence
Revised Adaptive Expertise Survey (2018)	Ferguson et al. (2018)	Limited evidence	Limited evidence	Moderate evidence
Individual Work Performance Questionnaire (2020)	Dåderman et al. (2020)	Strong evidence	Strong evidence	Moderate evidence
The Adaptability Scale (2021)	Van Dam et al. (2021)	Strong evidence	Strong evidence	Moderate evidence
Spanish adaptation of Charbonnier-Voirin and Roussel’s (2012) scale for measuring adaptive performance (2022)	Gorostiaga et al. (2022)	Strong evidence	Moderate evidence	Moderate evidence
*Instrument type: Supervisor evaluation*				
Job Adaptability Inventory (JAI) (2002)	Pulakos et al. (2002)	Strong evidence	Moderate evidence	Moderate evidence
*Instrument type: Design scenarios*				
Design scenarios (2006)	Walker et al. (2006)	Limited evidence	Limited evidence	Moderate evidence
*Instrument type: Mixed method*				
“Model for assessing teacher’s AE” (2016)	Yoon et al. (2019)	Limited evidence	Limited evidence	Limited evidence
Acquiring / creating teacher profiles on adaptive teaching expertise (2021)	Suh et al. (2023)	Strong evidence	Limited evidence	Moderate evidence
*Instrument type: Collegial verbalization*				
Collegial Verbalization (2018)	Axelsson et al. (2018)	Moderate evidence	Limited evidence	Moderate evidence

Strong evidence: multiple pieces of evidence

Moderate evidence: one piece of evidence

Limited evidence: no evidence or incomprehensible or incomplete evidence

Some articles lack an in-depth discussion of the research on the instrument's quality, and there is currently a substantial variation in the strength of evidence provided regarding the quality of the instruments.

## Discussion

The first aim of our study was to provide an overview of instruments that measure adaptive expertise and adaptive performance. Our review included 17 studies that described 19 instruments. Despite the extent of studies into the concepts of adaptive expertise and performance, research on measurement instruments of these concepts appears to be relatively scarce.

Our findings show a preference in the literature to report on self-evaluation instruments and job requirement instruments with Likert scales and on the development of those instrument types. Job evaluation and self-evaluation instruments are preferred for their usability, as they are easy to administer and rather easy to score. However, the other instrument types are genuinely relevant as well since some of the articles discuss alternative ways to measure adaptive expertise and adaptive performance. Although the authors of the design scenarios, mixed method instruments and collegial verbalization provide relatively little information on the relevant quality criteria, the instruments might offer possibilities for domain specific assessments that can also be used as (feedback) instruments in educational settings. These instruments are highly context-sensitive, closely aligned with daily practice and offer a different perspective on measuring adaptive expertise and adaptive performance. These instruments, however, will need to be further developed and rigorously studied to meet the quality criteria. To this end, alternative quality criteria may be needed with quality standards related to qualitative research, which differ from the established norms in the Standards that are based on a traditional psychometric view. If the instruments meet the quality criteria, the use of these latter instruments could enhance (becoming) professionals’ learning as the data from the instruments can contribute to formative assessment purposes in which gathering and providing information about a students’ current *performance* are central elements (Morris et al., 2021, p. 3). The instruments can play a role in fostering more formative and diversified assessment practices. This is needed, given the fact that traditional examinations (closed-book, individual, invigilated, time-constrained, summative, final, and high-stakes) have limited pedagogical value (French et al., 2023, p. 16). Therefore, we recommend investigating how the mix of instruments can be used to increase the validity and reliability of measuring adaptive expertise and performance.

Three instruments were identified specifically for (becoming) healthcare professionals in the domains of biomedical engineering, biomedical engineering design, and healthcare in general (Fisher et al., 2001). Ferguson et al. (2018) and Walker et al. (2006)). It is noteworthy that all these instruments were developed for the educational context, with two of them specifically within biomedical engineering education. Notably, the literature does not make a clear distinction regarding the target group an instrument is intended for (e.g., students or working professionals). This overlooks how adaptive expertise develops and, specifically, whether and how an instrument is tailored to a developing target group.

The second aim of our study was to bring greater clarity to the operationalisation of adaptive expertise and adaptive performance and, based on this, to refine their conceptualisation. Confirming findings of other studies in this topic (Cupido et al., 2022 and Pelgrim et al., 2022), this study encountered inconsistency in conceptualisation and operationalisation, meaning a unified definition and operationalisation of the concepts is lacking. Combined with different purposes, target groups and contexts to measure adaptive expertise and performance, this results in a variety of content and types of measurement instruments. Various instruments are derived from one another with some instruments using identical or nearly identical items for job evaluation, self-evaluation and/or supervisor evaluation. The theoretical foundation is frequently not elaborated upon in the articles. In our view, this appears to disregard the complexity of the concepts.

Based on the analysis of the subscales from all instruments, we were able to provide a further characterization of both adaptive expertise, which concerns individual characteristics, and adaptive performance, which relates to the work a professional performs. Notably, in this new categorization, 7 of the 8 themes that we classified under adaptive performance are the same as those indicated by Pulakos et al. in the Job Adaptability Inventory (Pulakos et al., 2000). This seems logical, as the validity and reliability of this instrument are supported by strong evidence, and several authors have based their own instruments on the Job Adaptability Inventory. The themes that we classified under adaptive expertise come from multiple instruments.

Through this analysis, based on operationalisations of the concepts, we took an initial step toward clarifying adaptive expertise and adaptive performance, demonstrating what these concepts entail in practice. As a next step, a comprehensive evolutionary concept analysis (Rodgers, 2000) might be useful as this approach offers a flexible and systematic method to explore and deeply understand concepts. This scoping review can serve as a foundational resource for this analysis, as it provides a detailed account of operationalisations, alongside literature focused on conceptual descriptions (e.g., Pelgrim et al., 2022 and Cupido, 2022).

Conceptually and operationally, we could not distinguish clear difference between the measurement instruments for healthcare settings and those for other work domains. Consistent with the literature, the instruments for use in these settings sometimes use similar conceptualisations and operationalisations and sometimes they differ. Our recommendation to researchers, educators, and HRD professionals in the healthcare domain would be to carefully assess which conceptualisation and operationalisation align with the specific needs of the users in the daily healthcare setting and to assess whether an instrument is mainly used to measure characteristics / traits (adaptive expertise) or work / tasks (adaptive performance).

The study's third aim was to evaluate the amount of evidence provided for the quality of the instruments. The job evaluation and self-evaluation instruments provide the most substantial proof for validity, reliability and fairness in testing, especially the Fleadapt Scale (Sony et al., 2015), the Individual Work Performance Questionnaire (Dåderman et al., 2020) and The Adaptability Scale (van Dam et al., 2021). The self-evaluation instruments in the healthcare domain are the least extensively described. Fisher et al.'s (2001) "An adaptive expertise Likert-scale" and Ferguson et al.'s (2018) Revised Adaptive Expertise Survey provided limited evidence regarding validity and reliability. The evidence is lacking, minimal, or insufficiently detailed. Certain instruments build on one another, inheriting their strengths and weaknesses. For instance, the Revised Adaptive Expertise Survey (Ferguson et al., 2018) is derived from “An Adaptive Expertise Likert scale” (Fisher et al., 2001), leading to shared shortcomings in validity and reliability. This may partly reflect limited study of these instruments or their relatively early stage in the development process.

Several instruments require further evaluation in their validity, reliability and fairness in testing before determining whether sufficient evidence exists to justify their continued use. Regarding the Design Scenarios instrument type, Walker's instrument also shows limited evidence on validity and reliability. However, it should be noted that the Standards' criteria are not entirely suitable for this type of instrument.

In conclusion, the evidence of validity, reliability and fairness in testing of the healthcare domain instruments is limited at this point in time and further validation of these instruments is recommended. The self-evaluation instruments can be further refined through this process. This also applies to the Design Scenarios, where we emphasize that, although this type of instrument currently lacks evidence of adequate validity and reliability, it can serve as an inspiration for developing situation-specific instruments within healthcare that are qualitative in nature and complement quantitative data.

Finally, healthcare professionals are advised to look beyond healthcare literature for knowledge development in the field of adaptive expertise and adaptive performance. This study shows that there are several instruments that may be sufficiently generic to be effectively utilized within the healthcare setting.

## Methodological limitations, strengths and implications

This scoping review is subject to some limitations. One limitation is the exclusive focus on international peer reviewed research articles. Even though we used two comprehensive databases, it is possible that we missed relevant publications that are only available through other databases. We limited our search to publications from between 1986 and 2023, and published in English. Though the included articles are valuable sources for measurement instruments, there may also be useful instruments that are not described in research articles or in articles just outside our criteria for inclusion.

Additionally, the evaluation of the quality of the instruments could have been more comprehensive if, in addition to the scientific publications, more documentation (e.g. manuals, test materials) had been considered. This could have particularly impacted the evaluation of reliability and fairness, as manuals and other test materials may provide more detailed information on these aspects. In our analysis, we primarily focused on the amount of evidence and, to a lesser extent, on the quality of this evidence. However, all articles are of sufficient quality, as they have been previously published in peer-reviewed journals.

One of the strengths of our scoping review is the thorough search which provided the data for the first overview of measurement instruments on adaptive expertise and adaptive performance that we are aware of. Besides, we contributed to the conceptualisation of adaptive expertise and adaptive performance by using the operationalisations of the authors which can serve as a valuable starting point for future research and practice. The entire process from the initial idea and the formulation of the research questions to the final synthesizing of the results has been conducted by an experienced and multidisciplinary team.

## Conclusion

This scoping review has provided insight into instruments that measure adaptive expertise and adaptive performance in (becoming) healthcare professionals, in both workplace and educational settings. An overview of 19 instruments is presented, representing six types of instruments. Although there is diversity in the utilization of terminology, conceptualisation and operationalisation of the terms adaptive expertise and adaptive performance, analysis of the subscales of the instruments provided further insight into what these terms entail in practice.

The study reveals a dominance of self-evaluation and job requirement instruments to measure adaptive expertise and performance, with varying quality. Other kinds of measurements, like design scenarios, mixed method instruments and collegial verbalization were poorly represented and evidence of their quality is limited.

Three measurement instruments are specifically developed for use in healthcare (education). The operationalisation and conceptualisation of adaptive expertise and adaptive performance in these instruments are generic and not specifically tailored for use within healthcare. The validity and reliability of these instruments require attention, necessitating follow-up research and development.

## Electronic supplementary material

Below is the link to the electronic supplementary material.

Supplementary file1 (XLSX 15 KB)

## Data Availability

Data is provided within the manuscript and supplementary information file.

## Appendix 1

### Used databases in web of science

1. Science Citation Index Expanded (SCI-EXPANDED)–1945-present
2. Social Sciences Citation Index (SSCI)–1956-present
3. Arts & Humanities Citation Index (AHCI)–1975-present
4. Conference Proceedings Citation Index—Science (CPCI-S)–1999-present
5. Conference Proceedings Citation Index—Social Science & Humanities (CPCI-SSH)–1999-present
6. Book Citation Index—Science (BKCI-S)–2005-present
7. Book Citation Index—Social Sciences & Humanities (BKCI-SSH)–2005-present
8. Emerging Sources Citation Index (ESCI)–2005-present
9. Current Chemical Reactions (CCR-EXPANDED)–1985-present
10. Index Chemicus (IC)–1993-present

### Used databases in scopus

https://www.elsevier.com/products/scopus/content.
